# Expression features of SOX9 associate with tumor progression and poor prognosis of hepatocellular carcinoma

**DOI:** 10.1186/1746-1596-7-44

**Published:** 2012-04-19

**Authors:** Xiaodong Guo, Lu Xiong, Ting Sun, Ruiyun Peng, Lin Zou, Haiyan Zhu, Jing Zhang, Hanwei Li, Jingmin Zhao

**Affiliations:** 1 Postgraduate Medical School of PLA, Beijing, 100853, China; 2 302 Hospital of PLA, Beijing, 100039, China; 3 Beijing Institute of Radiation Medicine, 27 Taiping Road, Beijing, 100850, China; 4 Navy General Hospital of PLA, Beijing, 100049, China; 5 PLA GENERAL HOSPITAL, Beijing, 100853, China

**Keywords:** Hepatocellular carcinoma, SOX9, Expression, Tumor progression, Prognosis

## Abstract

**Background:**

SOX9 as a member of the SOX (SRY [sex determining region Y] box) gene superfamily has been previously demonstrated to be a proto-oncogene in a variety of malignancies. However, the clinical significance of SOX9 expression in hepatocellular carcinoma (HCC) remains unclear. The aim of this study was to investigate the expression of SOX9 in HCC and determine its correlation with tumor progression and prognosis.

**Methods:**

One-hundred and thirty HCC patients who had undergone curative liver resection were selected and immunohistochemistry, Western blotting, and quantitative real time polymerase chain reaction (Q-PCR) were performed to analyze SOX9 expression in the respective tumors.

**Results:**

Immunohistochemistry, Western blotting, and Q-PCR consistently confirmed SOX9 overexpression in HCC tissues compared with their adjacent nonneoplastic tissues (P ≪ 0.01). Additionally, immunostaining showed more SOX9 positive cells in the higher tumor stage (T3 ~ 4) and tumor grade (G3) than in the lower tumor stage (T1 ~ 2, P = 0.03) and tumor grade (G1 ~ 2, P = 0.01), respectively. Moreover, HCC patients with high SOX9 expression were significantly associated with lower 5-year overall survival (P ≪ 0.01) and lower 5-year disease-free survival (P ≪ 0.01), respectively. The Cox proportional hazards model further showed that SOX9 over-expression was an independent poor prognostic factor for both 5-year disease-free survival (hazards ratio [HR] = 2.621, 95% confidence interval[CI] = 1.548-5.829, P = 0.01) and 5-year overall survival (HR = 3.825, CI = 1.638-7.612, P = 0.003) in HCC.

**Conclusion:**

Our data suggest for the first time that the overexpression of SOX9 protein in HCC tissues is of predictive value on tumor progression and poor prognosis.

**Virtual slides:**

The virtual slide(s) for this article can be found here: http://www.diagnosticpathology.diagnomx.eu/vs/9029740396926377.

## Introduction

Hepatocellular carcinoma (HCC) is one of the most frequent malignancies worldwide. Especially in China, it has become a major cause of cancer-related death [1]. As a highly aggressive solid tumor, HCC is characterized by fast infiltrating growth, early metastasis, high-grade malignancy, and poor prognosis. It is often secondary to hepatitis B virus (HBV) and hepatitis C virus (HCV) infections, both of which increase the risk of HCC 20-fold [2]. Curative therapies of surgical treatment, including hepatic resection and liver transplantation, improve the 2 short-term survival of HCC patients greatly. However, the prognosis for most patients remains poor because of multicentric recurrence and intrahepatic metastasis. The progression of HCC is a complicate process that associated with cumulative genomic alterations [3,4]. The aberrant gene expression, including oncogene upregulation and tumor suppressor downregulation, is responsible for the development of HCC. However, the molecular pathogenesis of HCC still remains unclear.

SOX9 (sex determining region Y [SRY] related high-mobility group box 9) is a member of the SRY (sex determining region Y) box gene superfamily [5]. As a transcriptional regulator, its expression has been observed in multiple tissues during embryogenesis, including cartilage, neural crest, notochord, kidney, pancreas, and endocardial cushions of the heart [6,7]. SOX9 takes an important part in chondrogenesis, male sex gonad or respiratory epithelium development, melanocyte differentiation, and the differentiation of Paneth cells in the gut [8,9]. Recently, SOX9 has also been demonstrated to be a proto-oncogene in a variety of malignancies [10-13]. For example, Wang et al. [14] detected the expression of SOX9 in prostate cancer cells contributes to tumor growth and invasion; In primary bladder tumours, Aleman et al. [15] found that SOX9 hypermethylation was present more than half of the cases and SOX9 hypermethylation was significantly associated with tumour grade and overall survival; Malki et al. [16] shown that the embryonic male prostaglandin D synthase/SOX9 pathway was expressed at both the RNA and protein levels in different types of human ovarian tumors, pointing to SOX9 as a possible diagnostic marker for ovarian carcinomas. However, the clinical significance of SOX9 expression in HCC remains unclear. The aim of this study was to investigate the expression of SOX9 in HCC and determine its correlation with tumor progression and prognosis.

## Materials and methods

### Patients and tissue samples

The study was approved by the Research Ethics Committee of 302nd Hospital of PLA, Beijing, China. Informed consent was obtained from all of the patients. All specimens were handled and made anonymous according to the ethical and legal standards.

A total of 130 patients with primary HCC who underwent a curative liver resection at the 302nd Hospital of PLA, Beijing, China, were included in this retrospective study. Tissues used in the study were retrieved from the tissue bank of the Department of Pathology in the 302nd Hospital of PLA. These patients were diagnosed as HCC between 2001 and 2006. None of the patients recruited in this study had chemotherapy or radiotherapy before the surgery. HCC diagnosis was based on World Health Organization (WHO) criteria. Tumor differentiation was defined according to the Edmondson grading system. Liver function was assessed using the Child-Pugh scoring system. Tumor staging was determined according to the sixth edition of the tumor-node-metastasis (TNM) classification of the International Union against Cancer. The clinicopathological features of 130 patients are summarized in Table I. In addition, 30 self-pairs of HCC specimens (5 TNM stage I, 8 TNM stage II, 12 TNM stage III, and 5 TNM stage IV) and adjacent nonneoplastic liver tissues were snap-frozen in liquid nitrogen and stored at −80°C following surgery for real-time quantitative RT-PCR assay and western blot analysis.

The median follow-up period was 8.6 years. Postoperative surveillance included routine clinical and laboratory examinations every third month, computed tomography scans of the abdomen, and radiographs of the chest every third month. After 5 years, the examination interval was extended to 12 months.

### Immunohistochemistry analysis

Immunohistochemical staining was carried out following the protocol of our previous study [17-19]. The primary antibody against SOX9: rabbit polyclonal antibody (Santa Cruz Biotechnology, Inc. USA), dilution 1:50. The specificity of the primary antibody has been validated by the previous studies of Müller et al. [20] and Lü et al. [21]. Secondary antibody for the detection of primary antibody: anti-rabbit IgG (Sigma, St. Louis, MO, USA). The negative controls were processed in a similar manner with PBS instead of primary antibody. Further, positive SOX9 expression confirmed by western blotting was used as positive controls for immunostaining.

Following a hematoxylin counterstaining, immunostaining was scored by two independent experienced pathologists, who were blinded to the clinicopathological parameters and clinical outcomes of the patients. The scores of the two pathologists were compared and any discrepant scores were trained through re-examining the stainings by both pathologists to achieve a consensus score. The number of positive-staining cells showing immunoreactivity in the nucleus for SOX9 in ten representative microscopic fields was counted and the percentage of positive cells was calculated. The percentage scoring of immunoreactive tumor cells was as follows: 0 (0%), 1 (1-10%), 2 (11-50%) and 3 (≫50%). The staining intensity was visually scored and stratified as follows: 0 (negative), 1 (weak), 2 (moderate) and 3 (strong). A final score was obtained for each case by multiplying the percentage and the intensity score. Therefore, tumors with a multiplied score exceeding 5 (median of total scores for SOX9) were deemed to be low expressions of SOX9; all other scores were considered to be high expressions of SOX9.

### Western blot

The Western blot protocol and semiquantitative analysis were carried out following the protocol of Xu et al [22]. SOX9 antibody (rabbit polyclonal antibody, dilution 1:50, Santa Cruz Biotechnology, Inc. USA) was used, and GAPDH antibody (CW0266, dilution 1:1,000, CoWin Biotech) was used as internal control.

### Quantitative RT-PCR

To measure the mRNA expression levels of SOX9, total RNA was extracted from frozen liver tissues using TriZol reagent (Invitrogen) following the manufacturer's instructions. Two micrograms of total RNA was subjected to reverse transcription to synthesize cDNA using the ProtoScript M-MuLV Taq RT-PCR Kit (New England Biolabs), according to the manufacture's instruction, followed by real-time PCR using the TransStart Green qPCR SuperMix (TransGen Biotech). The primer sequences of SOX9 were forward primer, 5'-CGA ACG CAC ATC AAG ACG A-3', reverse primer, 5'-AGG TGA AGG TGG AGT AGA GGC-3'. The transcription of GAPDH was used as an internal control for normalization. SOX9 expression levels were calculated relative to GAPDH using the delta-delta computed tomography method [23].

### Statistical analysis

The software of SPSS version13.0 for Windows (SPSS Inc, IL, USA) and SAS 9.1 (SAS Institute, Cary, NC) was used for statistical analysis. Fisher's exact test and the X^2^ test were performed to assess associations between SOX9 expression and clinicopathological parameters. The Kaplan-Meier method was used for survival analysis, and differences in survival were estimated using the log-rank test. A multivariate survival analysis was performed for all parameters that were significant in the univariate analyses using the Cox regression model. Differences were considered statistically significant when *P* was less than 0.05.

## Results

### Expression of SOX9 protein and mRNA in HCC

**Figure 1:**
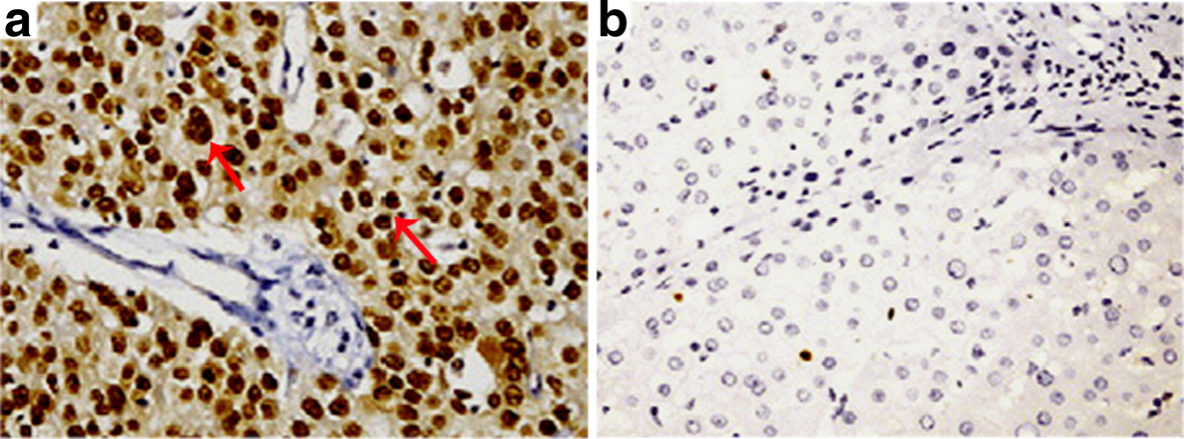
**SOX9 expression in hepatocellular carcinoma (HCC) and adjacent nonneoplastic liver tissues (Original magnification × 400). a**, SOX9 positive staining was indicated by numerous yellowish granules in the nucleus of HCC cells; **b**, SOX9 negative staining was seen in adjacent nonneoplastic liver tissues.

Immunohistochemical analysis revealed that SOX9 staining was mainly localized in the nucleus of HCC cells (Figure 1a). SOX9 expression was absent or sporadic in adjacent nonneoplastic liver tissues (Figure 1b). In addition, we found 98 (75.38%) of 130 HCC tissues with high SOX9 expression and 32 (24.62%) of 130 HCC tissues with low SOX9 expression, while 6 (4.62%) of 130 adjacent nonneoplastic liver tissues with high SOX9 expression and 124 (95.38%) of 130 adjacent nonneoplastic liver tissues with low SOX9 expression. Thus, the SOX9 immunostainings in HCC tissues were significantly higher than those in the adjacent nonneoplastic liver tissues (P ≪ 0.01).

**Figure 2:**
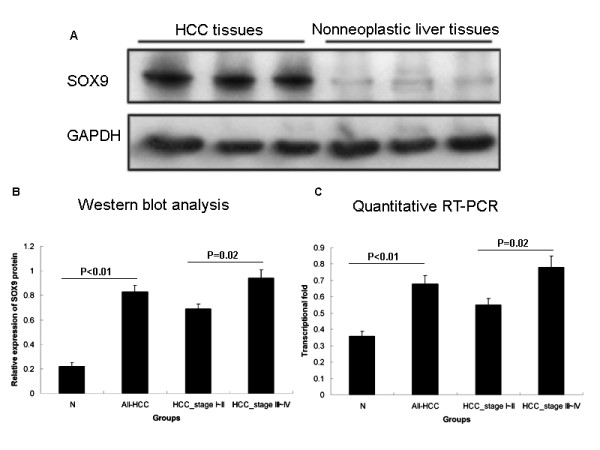
**Increased SOX9 protein and mRNA levels in hepatocellular carcinoma (HCC) with different TNM stages and adjacent nonneoplastic liver tissues.** (**a**) Representative Western blotting of SOX9 protein levels in HCC tissues and adjacent nonneoplastic liver tissues. (**b**) Semiquantitative Western blotting showed that the expression levels of SOX9 protein were significantly higher than those in adjacent nonneoplastic liver tissues (P ≪ 0.01). Additionally, the expression levels of SOX9 protein were increased with ascending tumor TNM stages. GAPDH was used as internal control. Means, standard deviation (SD), and P values were given (T test). (**c**) Quantitative RT-PCR assay showed significantly increased SOX9 mRNA level in HCC tissues compared with adjacent nonneoplastic liver tissues (P ≪ 0.01). Additionally, the expression levels of SOX9 mRNA were increased with ascending tumor TNM stages. GAPDH was used as internal control. Means, standard deviation (SD), and P values were given (Mann–Whitney test).

To confirm SOX9 protein expression by an independent method, Western blot analysis was performed using 30 self-pairs of HCC and adjacent nonneoplastic liver tissues. The distinct overexpression of SOX9 protein in HCC tissues compared with adjacent nonneoplastic liver tissues was also detected (P ≪ 0.01, Figure 2a and b), as well as significantly increased mRNA level by quantitative RT-PCR (P ≪ 0.01, Figure 2c). The expression levels of SOX9 protein and mRNA in HCC tissues with high stage (III-IV) were both significantly stronger than those with low stage (I-II; for protein and mRNA: both P = 0.02; Figure 2b and c).

### Association of SOX9 expression with the clinicopathological features of HCC

To evaluate whether SOX9 protein expression was associated with clinicopathological features of patients with HCC, we correlated immunohistochemical SOX9 staining results with T stage, tumor grade, presence of cirrhosis, underlying liver disease including alcohol abuse, viral hepatitis B and C, sex, and age (Table 1). As the results, we found that more SOX9 positive cells in the higher tumor stage (T3 ~ 4) and tumor grade (G3) than in the lower tumor stage (T1 ~ 2, P = 0.03) and tumor grade (G1 ~ 2, P = 0.01), respectively.

**Table 1:** **Clinicopathological features and the expression of SOX9 in 130** **hepatocellular carcinoma** **patients**

**Clinicopathological Features**	**Case**	**SOX9 expression frequency (n,%)**		**P**
		**High**	**Low**	
**Age (years)**				
≤50	72	55 (76.39)	17 (23.61)	NS
>50	58	43 (74.14)	15 (25.86)	
**Gender**				
Male	96	73 (76.04)	23 (23.96)	NS
Female	34	25 (73.53)	9 (26.47)	
**Tumor stage**				
T1	23	8 (34.78)	15 (65.22)	0.03
T2	40	25 (62.50)	15 (37.50)	
T3	52	50 (96.15)	2 (3.85)	
T4	15	15 (100.00)	0 (0)	
**Tumor grade**				
G1	31	18 (58.01)	13 (41.99)	0.01
G2	76	58 (76.32)	18 (23.68)	
G3	23	22 (95.65)	1 (4.35)	
**Growth pattern**				
Trabecular	101	78 (77.23)	23 (22.77)	NS
Nontrabecular	29	20 (68.97)	9 (31.03)	
**Cirrhosis**				
Yes	86	62 (72.09)	24 (27.91)	NS
No	44	36 (81.82)	8 (18.18)	
**Underlying liver disease**				
Alcoholic	25	18 (72.00)	7 (28.00)	NS
Hepatitis B	49	40 (81.63)	9 (18.37)	
Hepatitis C	35	28 (80.00)	7 (20.00)	
Unknown	21	12 (57.14)	9 (42.86)	

### Prognostic values of SOX9 expression in HCC

**Figure 3:**
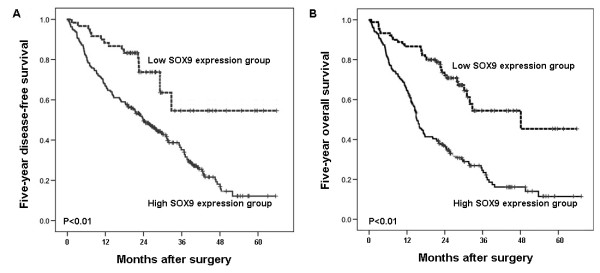
**Kaplan-Meier survival curves for SOX9 expression in** **hepatocellular carcinoma (HCC)** **patients**. The HCC patients with high SOX9 expression showed significantly shorter disease-free survival (P ≪ 0.01, **a**) and overall survival (P ≪ 0.01, **b**) rates than those with low SOX9 expression.

Five-year disease-free survival was observed in 30 (23.08%) patients, whereas in 100 (76.92%) patients, disease recurred, and 88 (67.69%) even died during a 5-year follow-up period. We observed a trend that 5-year disease-free survival in the group with high SOX9 expression was significantly poorer than that in the group with low SOX9 expression (P ≪ 0.01, log-rank test; Figure 3a). Additionally, the Kaplan-Meier plot of 5-year overall survival curves stratified by SOX9 expression was shown in Figure 3b. A significant relationship was found between SOX9 expression and 5-year overall survival (P ≪ 0.01, log-rank test, Figure 3). Futhermore, in a multivariate Cox model, including tumor size, tumor stage, tumor grading, presence of cirrhosis, gender, age, and SOX9 staining, we found that SOX9 expression was an independent poor prognostic factor for both 5-year disease-free survival (hazards ratio [HR] = 2.621, 95% confidence interval[CI] = 1.548-5.829, P = 0.01, Table 2) and 5-year overall survival (HR = 3.825, CI = 1.638-7.612, P = 0.003, Table 2) in HCC.

**Table 2:** **Multivariate survival analysis of five-year overall and disease-free survival in 130 patients with** **hepatocellular carcinoma**

**Features**	**Five-year overall survival**			**Five-year disease-free survival**		
	**HR**	**95% CI**	**P**	**HR**	**95% CI**	**P**
**Age**	1.132	0.316-3.516	0.192	1.536	0.322-3.736	0.125
**Gender**	1.191	0.345-3.857	0.136	1.559	0.357-3.831	0.131
**Tumor size**	1.931	0.685-4.056	0.063	1.953	0.615-4.273	0.062
**Tumor stage**	2.879	1.366-5.196	0.009	2.686	1.386-6.009	0.01
**Tumor grade**	1. 563	0.609-4.088	0.081	1.551	0.607-4.466	0.086
**Presence of cirrhosis**	1.919	0.738-4.102	0.063	1.921	0.793-4.219	0.062
**SOX9 expression**	3.825	1.638-7.612	0.003	2.621	1.548-5.829	0.01

## Discussion

In the present study, we provide the first analysis of SOX9 protein and mRNA expression in human HCC tissue and its association with patient clinical outcome. SOX9 immunoreactivity was significantly increased in a substantial proportion of HCC cases compared with their adjacent nonneoplastic liver tissue. The overexpression of SOX9 was observed in tumor tissues with higher tumor stage and higher tumor grade. Additionally, our investigation reveals that high SOX9 expression is associated with a significant trend toward both poorer disease-free survival and poorer overall survival. Our study further confirms that high SOX9 expression independently predicts a higher risk of disease relapse or death after multivariate adjustment for other prognostic factors.

Members of SOX gene family share homology with the high-mobility group box of the sex-determining region Y (SRY), which encodes transcription factors that bind to high-mobility group domains of DNA [24]. SOX9 belongs to the subgroup of SOX E genes, which play vital roles in the regulation of the differentiation of astrocytes, oligodendrocytes, and Schwann cells [25]. SOX9 is involved in the development of multiple tissues and in maintaining the stem cell compartments in adult tissues [26]. Mutations in the SOX9 gene may result in autosomal XY sex reversal and in campomelic dysplasia, a syndrome with severely malformed skeleton [27]. Recent studies have demonstrated the direct roles for SOX9 in tumorigenesis. In digestive system tumors, Jiang et al. [28] found that SOX9-transfected cells injected into severe combined immunodeficient mice show markedly stronger tumorigenicity, whereas SOX9-knockdown cells injected into severe combined immunodeficient mice show significantly attenuated tumorigenicity in mice. Sashikawa et al. [29] then detected the expression of SOX9 in human intestinal metaplasia and gastric carcinoma. Liu et al. [30] further demonstrated that SOX9 expression significantly increased from nonneoplastic lesions to gastric neoplastic lesions, which might promote the tumor progression of gastric carcinoma. On the other hand, Jay et al. [31] found that the overexpression of SOX9, a novel intestinal crypt transcription factor, may inhibit carcinoembryonic antigen expression and may induce apoptosis in a human colon carcinoma cell line. In human colorectal cancer tissues, Lü et al. [21] also detected the overexpression of SOX9, and further demonstrated that the detection of SOX9 expression might contribute to predicting clinical outcomes for patients with this tumor. However, the role of SOX9 in HCC remains to be elucidated. In this study, our data may offer new insight into SOX9 that is potentially important in the progression of HCC, as well as new prognostic factor for HCC. As the 130 cases of the present study were all Chinese population, the results reported here should be further confirmed in other populations.

In conclusion, our study suggests that SOX9 is overexpressed in HCC tissues compared with their benign counterparts. To the best our knowledge, this is the first study evaluating the expression levels of SOX9 mRNA and protein in HCC tissues and its association with clinicopathologic parameters. Especially, the most important finding of this study is that SOX9 also is a novel and potential factor for predicting the poorer prognosis of HCC patients after surgery. Further studies are needed to investigate the precise function of SOX9 in the progression of HCC.

## Competing interests

The authors declare that they have no competing interests.

## Authors' contributions

Guo XD, Xiong L, Li HW and Zhao JM: participated in study design and coordination, analysis and interpretation of data, material support for obtained funding, and supervised study; Sun T, Peng RY, and Zou L: help to translated and edit the paper; Zhu HY and Zhang J: carry out part of the experiments. All authors read and approved the final manuscript.

## References

[B1] Li J Jiang X Loss of runt-related transcription factor 3 expression associated with human hepatocellular carcinoma progression and prognosis Asian Pac J Cancer Prev 2011 12 2285 2290 22296371

[B2] Zhong C Wei W Su XK Li HD Xu FB Guo RP Serum and tissue vascular endothelial growth factor predicts prognosis in hepatocellular carcinoma patients after partial liver resection Hepatogastroenterology 2012 59 93 97 22251524 10.5754/hge10638

[B3] Lin W Chen YL Jiang L Chen JK Reduced expression of chemerin is associated with a poor prognosis and a lowed infiltration of both dendritic cells and natural killer cells in human hepatocellular carcinoma Clin Lab 2011 57 879 885 22239017

[B4] Dettmer M Itin P Miny P Gandhi M Cathomas G Willi N Giant ectopic liver, hepatocellular carcinoma and pachydermia-a rare genetic syndrome? Diagn Pathol 2011 6 75 21831298 10.1186/1746-1596-6-75 PMC3162532

[B5] Foster JW Dominguez-Steglich MA Guioli S Kwok C Weller PA Stevanović M Weissenbach J Mansour S Young ID Goodfellow PN Campomelic dysplasia and autosomal sex reversal caused by mutations in an SRY-related gene Nature 1994 372 525 530 7990924 10.1038/372525a0

[B6] Wagner T Wirth J Meyer J Zabel B Held M Zimmer J Pasantes J Bricarelli FD Keutel J Hustert E Wolf U Tommerup N Schempp W Scherer G Autosomal sex reversal and campomelic dysplasia are caused by mutations in and around the SRY-related gene SOX9 Cell 1994 79 1111 1120 8001137 10.1016/0092-8674(94)90041-8

[B7] Bi W Huang W Whitworth DJ Deng JM Zhang Z Behringer RR de Crombrugghe B Haploinsufficiency of SOX9 results in defective cartilage primordia and premature skeletal mineralization Proc Natl Acad Sci USA 2001 98 6698 6703 11371614 10.1073/pnas.111092198 PMC34415

[B8] Hersmus R Kalfa N de Leeuw B Stoop H Oosterhuis JW de Krijger R Wolffenbuttel KP Drop SL Veitia RA Fellous M Jaubert F Looijenga LH FOXL2 and SOX9 as parameters of female and male gonadal differentiation in patients with various forms of disorders of sex development (DSD) J Pathol 2008 215 31 38 18348162 10.1002/path.2335

[B9] Knower KC Kelly S Ludbrook LM Bagheri-Fam S Sim H Bernard P Sekido R Lovell-Badge R Harley VR Failure of SOX9 regulation in 46XY disorders of sex development with SRY, SOX9 and SF1 mutations PLoS One 2011 6 e17751 21412441 10.1371/journal.pone.0017751 PMC3055899

[B10] Ling S Chang X Schultz L Lee TK Chaux A Marchionni L Netto GJ Sidransky D Berman DM An EGFR-ERK-SOX9 signaling cascade links urothelial development and regeneration to cancer Cancer Res 2011 71 3812 3821 21512138 10.1158/0008-5472.CAN-10-3072 PMC3107391

[B11] Chakravarty G Moroz K Makridakis NM Lloyd SA Galvez SE Canavello PR Lacey MR Agrawal K Mondal D Prognostic significance of cytoplasmic SOX9 in invasive ductal carcinoma and metastatic breast cancer Exp Biol Med (Maywood) 2011 236 145 155 21321311 10.1258/ebm.2010.010086

[B12] Krahl D Sellheyer K Basal cell carcinoma and pilomatrixoma mirror human follicular embryogenesis as reflected by their differential expression patterns of SOX9 and β-catenin Br J Dermatol 2010 162 1294 1301 20105172 10.1111/j.1365-2133.2010.09630.x

[B13] Afonja O Raaka BM Huang A Das S Zhao X Helmer E Juste D Samuels HH RAR agonists stimulate SOX9 gene expression in breast cancer cell lines: evidence for a role in retinoid-mediated growth inhibition Oncogene 2002 21 7850 7860 12420222 10.1038/sj.onc.1205985

[B14] Wang H Leav I Ibaragi S Wegner M Hu GF Lu ML Balk SP Yuan X SOX9 is expressed in human fetal prostate epithelium and enhances prostate cancer invasion Cancer Res 2008 68 1625 1630 18339840 10.1158/0008-5472.CAN-07-5915

[B15] Aleman A Adrien L Lopez-Serra L Cordon-Cardo C Esteller M Belbin TJ Sanchez-Carbayo M Identification of DNA hypermethylation of SOX9 in association with bladder cancer progression using CpG microarrays Br J Cancer 2008 98 466 473 18087279 10.1038/sj.bjc.6604143 PMC2361432

[B16] Malki S Bibeau F Notarnicola C Roques S Berta P Poulat F Boizet-Bonhoure B Expression and biological role of the prostaglandin D synthase/SOX9 pathway in human ovarian cancer cells Cancer Lett 2007 255 182 193 17532558 10.1016/j.canlet.2007.04.007

[B17] GUO XD XIONGL ZOU L ZHAO JM Upregulation of bone morphogenetic protein 4 is associated with poor prognosis in patients with hepatocellular carcinoma Pathology & Oncology Research In press10.1007/s12253-011-9488-2 22350792

[B18] Schmilovitz-Weiss H Tobar A Halpern M Levy I Shabtai E Ben-Ari Z Tissue expression of squamous cellular carcinoma antigen and Ki67 in hepatocellular carcinoma-correlation with prognosis: A historical prospective study Diagn Pathol 2011 6 121 22151825 10.1186/1746-1596-6-121 PMC3286417

[B19] Hong H Patonay B Finley J Unusual reticulin staining pattern in well-differentiated hepatocellular carcinoma Diagn Pathol 2011 6 15 21338527 10.1186/1746-1596-6-15 PMC3049127

[B20] Müller P Crofts JD Newman BS Bridgewater LC Lin CY Gustafsson JA Ström A SOX9 mediates the retinoic acid-induced HES-1 gene expression in human breast cancer cells Breast Cancer Res Treat 2010 120 317 326 19322650 10.1007/s10549-009-0381-6

[B21] Lü B Fang Y Xu J Wang L Xu F Xu E Huang Q Lai M Analysis of SOX9 expression in colorectal cancer Am J Clin Pathol 2008 130 897 904 19019766 10.1309/AJCPW1W8GJBQGCNI

[B22] Xu MZ Yao TJ Lee NP Ng IO Chan YT Zender L Lowe SW Poon RT Luk JM Yes-associated protein is an independent prognostic marker in hepatocellular carcinoma Cancer 2009 115 4576 4585 19551889 10.1002/cncr.24495 PMC2811690

[B23] Livak KJ Schmittgen TD Analysis of relative gene expression data using real-time quantitative PCR and the 2(−Delta Delta C(T)) Method Methods 2001 25 402 408 11846609 10.1006/meth.2001.1262

[B24] Kent J Wheatley SC Andrews JE Sinclair AH Koopman P A male specific role for SOX9 in vertebrate sex determination Development 1996 122 2813 2822 8787755 10.1242/dev.122.9.2813

[B25] Spokony RF Aoki Y Saint-Germain N Magner-Fink E Saint-Jeannet JP The transcription factor SOX9 is required for cranial neural crest development in Xenopus Development 2002 129 421 432 11807034 10.1242/dev.129.2.421

[B26] Blache P van de Wetering M Duluc I Domon C Berta P Freund JN Clevers H Jay P SOX9 is an intestine crypt transcription factor, is regulated by the Wnt pathway, and represses the CDX2 and MUC2 gene J Cell Biol 2004 166 37 47 15240568 10.1083/jcb.200311021 PMC2172132

[B27] Liang B Cotter MM Chen D Hernandez CJ Zhou G Ectopic Expression of SOX9 in Osteoblasts Alters Bone Mechanical Properties Calcif Tissue Int 2012 90 76 89 22143895 10.1007/s00223-011-9550-9 PMC3272153

[B28] Jiang SS Fang WT Hou YH Huang SF Yen BL Chang JL Li SM Liu HP Liu YL Huang CT Li YW Jang TH Chan SH Yang SJ Hsiung CA Wu CW Wang LH Chang IS Upregulation of SOX9 in lung adenocarcinoma and its involvement in the regulation of cell growth and tumorigenicity Clin Cancer Res 2010 16 4363 4373 20651055 10.1158/1078-0432.CCR-10-0138

[B29] Sashikawa Kimura M Mutoh H Sugano K SOX9 is expressed in normal stomach, intestinal metaplasia, and gastric carcinoma in humans J Gastroenterol 2011 46 1292 1299 21861142 10.1007/s00535-011-0443-5

[B30] Liu JN Shang Guan YM Qi YZ Wang HB Zhang TG Zhou CJ The evaluation of SOX9 expression and its relationship with carcinoembryonic antigen-related cell adhesion molecule 1 in gastric neoplastic and nonneoplastic lesions Ann Diagn Pathol 2011 In press 10.1016/j.anndiagpath.2011.10.003 22209504

[B31] Jay P Berta P Blache P Expression of the carcinoembryonic antigen gene is inhibited by SOX9 in human colon carcinoma cells Cancer Res 2005 65 2193 2198 15781631 10.1158/0008-5472.CAN-04-1484

